# The Standard Opaque Meal for X-Ray Examination

**Published:** 1915-04-24

**Authors:** 


### THE STANDARD OPAQUE MEAL FOR X-RAY EXAMINATION.

### The Cheapness of Barium Sulphate.

For a considerable time radiologists have felt the need, for the purpose of a reliable comparison of results in various hands, of a standard opaque meal, so that nothing more need be taken into account than the appearances presented by the plate. Accordingly, some eighteen months ago, the Electro-Therapeutic Section of the Royal Society of Medicine appointed a sub-committee to go into the matter, and report to the section. This body consisted of Dr. Reginald Morton, Dr. A. E. Barclay, Dr. A. F. Hertz, Dr. A. C. Jordan, and Mr. S. Gilbert Scott, and at the meeting of the section on Friday, 16th inst., Dr. Morton presented their report, in which the following are the chief points:—

The Sub-Committee's Report.

The standard meal can consist of either bread-and-milk, or porridge, and the bulk recommended is about half a pint. The meal should be mixed with 2 oz. to 3 oz. of barium sulphate or bismuth oxychloride, and the patient should receive it when the stomach is as empty as possible. No aperient, or other medicine, should be taken within thirty-six hours of the first examination, and if the bowels are not opened naturally, an enema should be given on the morning of the examination. In preparing the bread-and-milk, 2 oz. of white bread, without crust, should be cut into small cubes, and after 8 oz. of ordinary or malted milk has been boiled in a separate vessel and mixed with 2 oz. of the bismuth or barium, the mixture should be poured over the bread, sugar being added to suit the taste. In the case of porridge 7 oz. of finest oatmeal are mixed with 2 oz. bismuth or barium, and milk should be added to bring the total bulk to 10 oz., sugar here also being added to taste.

At the request of Dr. Jordan, a minority report was also submitted, for that gentleman held that the meals set out were not suitable for chronic intestinal stasis. In the case of patients suffering from this latter condition, he recommended an emulsion containing 4 oz. carbonate of bismuth, with sugar of milk and water, to be taken within two hours after an ordinary breakfast, no purge or enema having been given.

The Discussion.

The section then proceeded to discuss the standard meal. Dr. Hertz thought a note should be added directing attention to the much greater cheapness of barium sulphate for hospital use, with practically equal effect from the radiographic standpoint. By such substitution Guy's Hospital had saved some £50 per annum. This was supported by Dr. Barclay.

Dr. Metcalfe thought some indications should be given in order to render the opaque meal more palatable, by the addition of flavourings. He thought chocolate or cocoa was relished by most. Not only would it then be more acceptable to patients whose digestion was none too robust, but there would be less chance of erroneous readings due to repugnance to the meal.

Dr. Jordan agreed with Dr. Metcalfe, but pointed to the difficulty that one would need to know in advance what was the particular patient's preference. In reference to the minority report, the emulsion he recommended would render opaque the meal already in the stomach.

Dr. Bailey put in a plea for cornflour, which many people would prefer to bread-and-milk or porridge.

The President, Dr. Ironside Bruce, expressed the hope that members of the section would follow out the suggestions of the sub-committee. Flavouring had purposely been left an open matter for each practitioner.

Dr. Reginald Morton replied, pointing out that the aim of the sub-committee was to lay down a basis, not to urge details. He agreed that such a meal should be rendered as little distasteful to the patient as possible. He did not see why oxychloride of bismuth should not be left out altogether. He believed that in some of his cases barium hastened the digestion of the meal, but he would be glad if the meeting would agree to adopt barium exclusively for all cases.

It was agreed to recommend and use barium sulphate only for these cases, and the reports were accepted. Reference was made to the loss sustained by the death of Dr. H. Lewis Jones, and a resolution of sympathy with his widow was carried by members rising in their places.

